# An updated review of Cucurbitacins and their biological and pharmacological activities

**DOI:** 10.17179/excli2015-283

**Published:** 2015-05-05

**Authors:** Sun Ok Chung, Yong Joo Kim, Sang Un Park

**Affiliations:** 1 Department of Biosystems Machinery Engineering, Chungnam National University, 99 Daehak-ro, Yuseong-gu, Daejeon 305-764, Korea; 2 Department of Crop Science, Chungnam National University, 99 Daehak-ro, Yuseong-gu, Daejeon 305-764, Korea

## Dear Editor,

Cucurbitacins (Cus) are a class of highly oxidized tetracyclic triterpenoids that confer a bitter taste to cucurbits such as cucumber, melon, watermelon, squash, and pumpkin. To date, a large number of Cus and Cu-derived compounds have been isolated from the Cucurbitaceae family and from other species of the plant (Alghasham, 2013[1]; Shang et al., 2014[2]). 

Cus have a range of biological and pharmacological activities that first attracted attention in the 1960s (Chen et al., 2012[3]). Cucurbitacin B (CuB) and Cucurbitacin E (CuE) have been particularly widely studied (Lan et al., 2013[4]). Recent reports have demonstrated that CuE has growth-inhibitory effects in the proliferation of many cancer cells such as bladder cancer, hepatocellular carcinoma, pancreatic cancer, breast cancer, and leukemia (Dong et al., 2010[5]; Sörensen et al., 2012[6]). CuB has been shown to have antimicrobial and anti-inflammatory activity. However, most reports on CuB focus on its anticancer activity. CuB inhibits the growth of human malignant cells, both in vitro and in vivo, and has been shown to be effective against breast cancer, head and neck squamous cell carcinoma, pancreatic cancer, hepatocellular carcinoma, osteosarcoma, and myeloid leukemia (Duangmano et al., 2010[7]; Kausar et al., 2013[8]; Guo et al., 2014[9]). 

Consequently, natural and semisynthetic Cus are proposed as a promising source for the development of new drugs for the prevention and treatment of various cancers. Here, we summarize key recent studies that have evaluated the biological and pharmacological activities of Cu and its derivatives (Table 1(Tab. 1)). 

References in Table 1: Zhang et al., 2014[10]; Jacquot et al., 2014[11]; Kong et al., 2014[12]; Guo et al., 2014[13]; Feng et al., 2014[14]; Hsu et al., 2014[15]; Gupta and Srivastava, 2014[16]; Gao et al., 2014[17]; Yuan et al., 2014[18]; Wang et al., 2014[19]; Ma et al., 2014[20]; Seo et al., 2014[21]; Kim et al., 2013[22]; Johnson et al., 2013[23]; Song et al., 2013[24]; Lan et al., 2013[4]; Hung et al., 2013[25]; Spear et al., 2013[26]; He et al., 2013[27]; Qiao et al., 2013[28]; Kausar et al., 2013[8]; Abbas et al., 2013[29]; Aribi et al., 2013[30]; Duangmano et al., 2012[31]; Zhang et al., 2012[32].

## Acknowledgements

This research was supported by Agriculture, Food and Rural Affairs Research Center Support Program, Ministry of Agriculture, Food and Rural Affairs.

## Figures and Tables

**Table 1:**
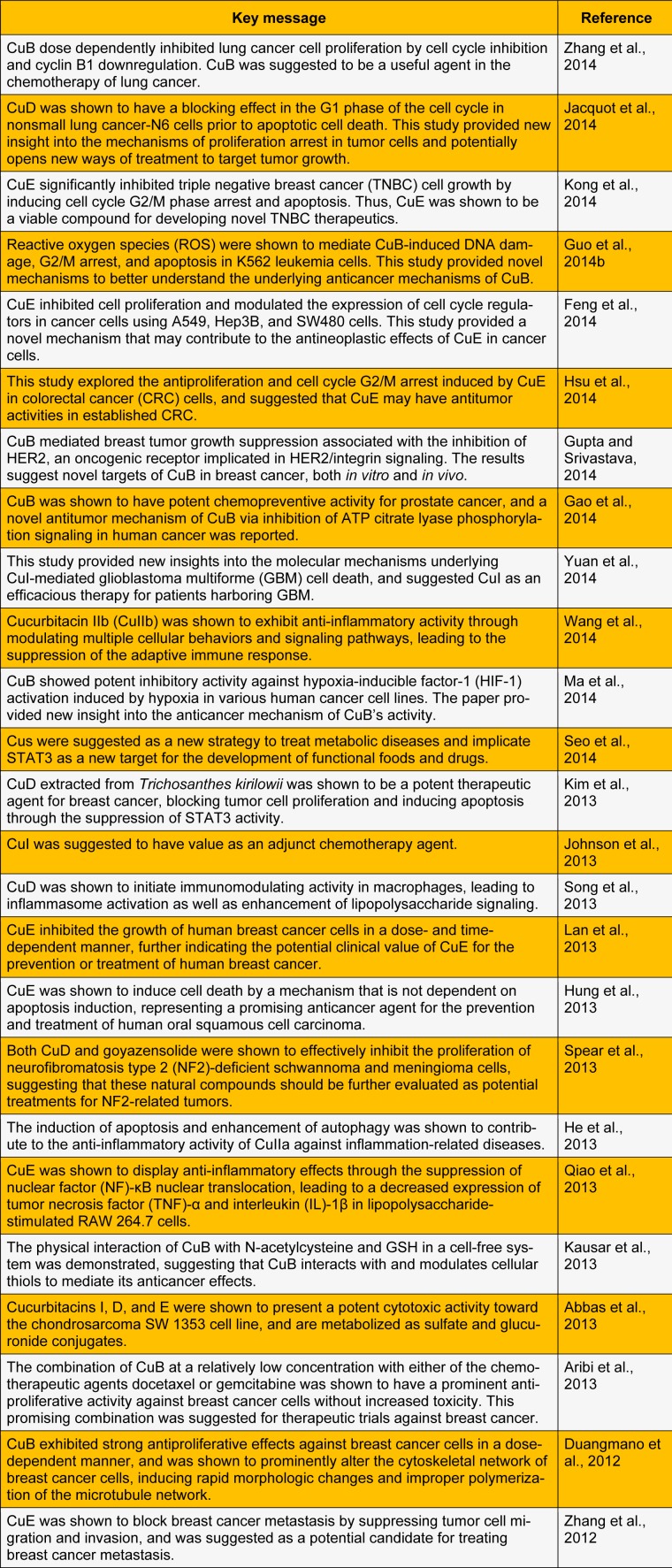
Recent studies on Cus and their biological and pharmacological activities
